# Is Mushy Tuna Syndrome a Growing Problem for the Tuna Industry?

**DOI:** 10.3390/foods12193590

**Published:** 2023-09-27

**Authors:** Soni Maria Jacob Peter, Simone P. Blomberg, Matthew H. Holden, Louwrens C. Hoffman, Ian R. Tibbetts

**Affiliations:** 1 School of the Environment, The University of Queensland, Brisbane 4072, Australia; s.peter@uq.edu.au (S.M.J.P.); s.blomberg1@uq.edu.au (S.P.B.); i.tibbetts@uq.edu.au (I.R.T.); 2 School of Mathematics and Physics, The University of Queensland, Brisbane 4072, Australia; m.holden1@uq.edu.au; 3 Centre for Nutrition and Food Sciences, Queensland Alliance for Agriculture and Food Innovation (QAAFI), The University of Queensland, Brisbane 4072, Australia; 4 Department of Animal Sciences, University of Stellenbosch, Stellenbosch 7600, South Africa

**Keywords:** environment, food loss and waste, food security, fish texture, Mushy Tuna Syndrome, skipjack tuna, sustainability

## Abstract

Reducing food loss and waste is crucial for a sustainable global food system and an efficient use of natural resources. Fast-growing tuna provides a key contribution to global nutrition targets; however, reports suggest that an appreciable proportion of the catch is lost from its value chain due to flesh quality issues, one of which is Mushy Tuna Syndrome (MTS). MTS-affected tuna flesh becomes soft and pasty, unfit for canning or human consumption, resulting in high wastage of partially processed material. We investigated the prevalence of MTS globally by surveying the tuna industry using a questionnaire. Of the responses from 32 companies across 14 nations, 97% acknowledged MTS as an issue that predominantly affects skipjack (*Katsuwonus pelamis*) tuna. The cost of rejects reported by participants from 2017 to 2019 varied greatly, from less than 1000 USD per year to over 1 million USD. The median cost was over 60,000 USD and the average rejection rate was 1.8%. The occurrence of MTS was noted to be seasonal, mainly in the summer months. More than half of the respondents who experience MTS reported an increasing trend of occurrence. Industry perceptions suggest MTS causes are associated with environmental, physiological, and biological factors. The survey results highlight that MTS is prevalent in the industry and demonstrate the need to identify amelioration strategies for the fishers and processors to minimise loss and maximise resource efficiency.

## 1. Introduction

The United Nation’s Sustainable Development Goal 12.3 calls for accelerated action to reduce food loss and waste, with a target to halve food waste by 2030 [1]. Food loss and waste are defined as “a reduction in food quantity and quality” [2] and refer to “food lost or wasted in the part of food chains leading to edible products going to human consumption” [3]. In the context of fish as food for more than 3.3 billion people globally, fish provides 20% of their average per capita intake of animal protein, making fisheries central to achieving food security [4]. However, fish is a highly perishable food, which is especially vulnerable to loss [5]. Globally, it is estimated that 30 to 35% of fishery and aquaculture production is lost or wasted annually [6].

The tuna industry is a particularly important example of the potential benefits that can be achieved through reduced seafood waste. Tunas (Family Scombridae, Tribe Thunnini) are important species as they not only play an important role in the ocean ecosystem as being one of the top predators [7], but are also an affordable source of nutritious and high-quality protein [6]. They are a major resource for potential economic development and food security for many island nations [8]. Tuna contributes over 40 billion USD to the global economy annually [9], with global catch steadily increasing [1,9]. This increase is partially driven by the demand for canned tuna, as a simple and convenient seafood choice, especially in the United States and Europe [10]. In 2018, canneries produced 4.1 million metric tonnes of tuna, which had an end value of nearly 26 billion USD [11].

**Figure 1:**
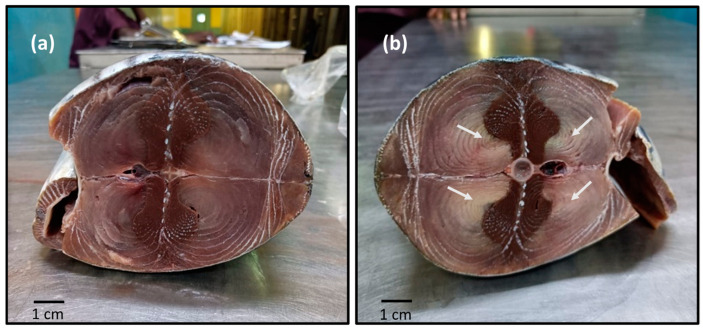
Cross section of defrosted skipjack tuna showing the difference in the colouration of muscles close to the backbone between normal skipjack tuna (**a**) and skipjack tuna muscle with Mushy Tuna Syndrome, which shows pale white discolouration in the region closest to the backbone of the fish (**b**) (indicated by white arrows). [Image by SMJP].

Despite the high societal and economic value of the tuna industry, a considerable amount of tuna catch is lost due to various flesh quality issues. Some of the flesh quality issues known to the tuna industry are “decomposition and spoilage associated with high histamine” [12,13,14], “burnt tuna” [15,16], “jelly flesh” [17,18], “honeycombing” [13,14,19], and “Mushy Tuna Syndrome” (MTS) [20]. MTS is a post-harvest textural quality defect in tuna wherein pronounced proteolytic softening of tuna meat is evident during processing, resulting in a canned product with an unacceptably mushy texture [20]. While MTS may affect raw fish [20], it mostly causes a high number of rejections and the downgrading of processed fish for canning [21]. MTS can be identified as a white, soft exudate in the region closest to the backbone of the fish and is found predominantly in skipjack tuna (*Katsuwonus pelamis*) [20] (Figure 1a,b). An unpublished study by Kaneko and Nakamura [22] was conducted on a single voyage of a purse seiner in Pago Pago in 1996, which recommended further study with a larger sample for understanding the predisposing factors of MTS. While Stagg et al., [20], found that raw fish with severe MTS showed higher levels of proteolysis, no research has investigated factors that contribute to the occurrence of MTS in raw tuna. This is particularly important because skipjack tuna is the dominant variety of tuna, making up 57% of global catch, and is reportedly sold in nearly every region of the world [11]. Skipjack is also the most sustainable for tuna fishery as the species is very fast-growing and relatively short-lived [23,24], showing a rapid turnover of their population [24].

Therefore, given the importance of skipjack tuna, assessing the causes of MTS, its magnitude, and its impacts, are essential steps towards being able to effectively address the problem of post-harvest loss in a tuna value chain, which broadly includes harvesting, storage, processing, and marketing. While there are reports on the occurrence of MTS [20,21,22], information is lacking on the extent of this issue in the tuna industry. Thus, we conducted a survey to collect and analyze information from the industry to understand the scope of MTS, evaluate trends in its occurrence, and estimate its economic impact on the industry. Here, we highlight the findings from the tuna industry survey and form a knowledge base with which to inform further research on the drivers of MTS in skipjack tuna.

## 2. Materials and Methods

We conducted a questionnaire-based tuna industry survey from August 2020 to December 2020. Given the global nature of the tuna industry [1], the survey was designed with a participatory approach to get respondents from different geographical locations. The questionnaire was distributed by email to potential respondents through tuna industry stakeholders and government officers, and by directly contacting potential participants. The questionnaire collected profiling information from each respondent (scope of business, species handled, number of employees, tonnage per day, products, and main markets by country), followed by the incidence of MTS (occurrence of MTS, species, quantity and cost of rejects, trend, potential causes, consequences to business, and handling of rejected material). Respondents were also asked to provide data on fishing methods and fishing grounds, as well as their comments on the causes and impact of MTS. Respondents completed a consent form, which included a note on the anonymity of data for publication purposes and advice not to respond to questions they held to be confidential. Data were de-identified for analysis, and analyses were performed using R [25]. This study was approved by the University of Queensland Human Research Ethics Committee (Ethics reference 2020001461).

## 3. Results

### 3.1. Survey Response and Respondent Characteristics

A total of 32 participants from 14 countries completed the survey from across Asia (59%), Oceania (28%), South America (9%), and Africa (3%). No responses were received from five potential participants who were from Asia (three), Oceania (one) and North America (one). Of the 32 participants, 67% reported the production of canned tuna, 81% frozen precooked tuna loins, and 56% whole, round, frozen fish. Two participants operated their own tuna fishing vessels. Analysis of the responses showed that 59% produced more than 100 MT tuna per day, with an average workforce of greater than 2300, followed by 31% who produced less than 100 MT tuna per day with an average workforce of over 500. Skipjack and yellowfin (*Thunnus albacares*) tunas were processed by 97% of the respondents, followed by bigeye tuna (*Thunnus obesus*) (66%), albacore tuna (*Thunnus alalunga*) (41%), bonito (*Sarda sarda*) (13%), and tonggol (*Thunnus tonggol*) (6%) (Table 1).

**Figure 2:**
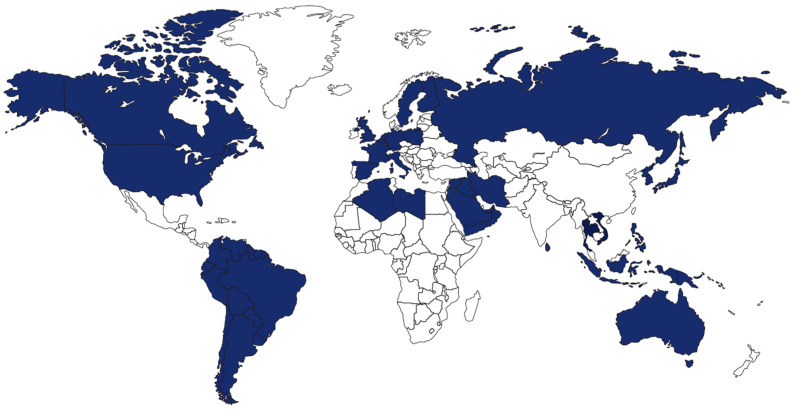
Geographic distribution of the main tuna product markets by country, as reported by the participants of the survey (n = 32).

The export market serviced by respondents covered a large geographic region, with over 35 countries in the Americas, Asia, European Union, Eurasian Economic Union (EAEU), the Middle East, and the Pacific (Figure 2).

### 3.2. Occurrence of Mushy Tuna Syndrome

**Figure 3:**
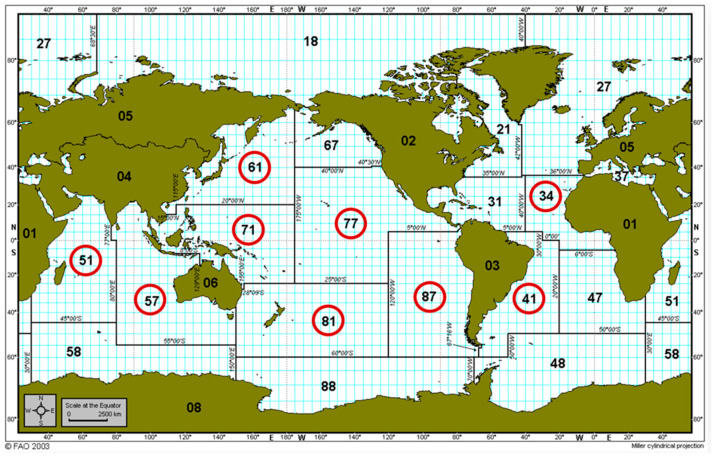
FAO, fishing zone map; encircled are the fishing zones where the respondents who experienced MTS received skipjack tuna from.

Out of the 29 participants who responded ‘Yes’ to MTS incidence, 97% (28/29) experienced MTS in skipjack tuna intended for canning, followed by yellowfin (10%), bigeye (10%), and albacore (3%). The survey found that 76% of the respondents who experienced MTS caught their tuna from FAO zone 71 and 38% from FAO zone 77, which lie in the Western Central Pacific Ocean and Eastern Central Pacific Ocean, respectively. Two participants who only caught tuna from FAO zone 51 experienced MTS in skipjack tuna. Skipjack was also caught from FAO zones 57, 61, 81, 34, 87, and 41 (Figure 3).

### 3.3. Rejection of Skipjack Tuna Due to Mushy Tuna Syndrome

**Figure 4:**
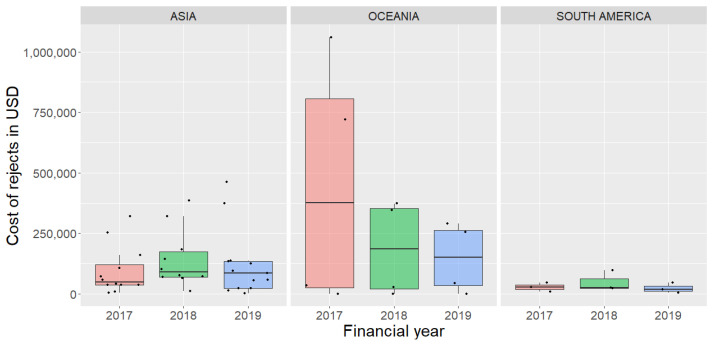
Boxplot of cost of rejection in USD due to Mushy Tuna Syndrome reported by participants in an industry survey Asia (n = 12), Oceania (n = 4), and South America (n = 3).

Data requested from the participants included the quantity of MTS rejects, total quantity processed, and an estimate of the cost of rejection for skipjack tuna because of MTS for the years 2017, 2018, and 2019. Data were provided by 72% (21/29) of the participants who experienced MTS in skipjack tuna. We calculated the percentage of MTS rejects and its cost for participants (Asia n = 12, Oceania n = 4 and South America n = 3) who provided data on the quantity of MTS skipjack, total skipjack tuna processed, and cost of rejection. Participants from Asia and Oceania had yearly rejection rates with a median of less than 2% and maximum rejects of over 6% in a year. The median cost was over 60,000 USD and the average rejection rate was 1.8%. The cost of rejects reported by participants ranged from less than 1000 USD per annum to over 1 million USD, based on the scale of their operation (Figure 4). To the question on the trend of MTS incidence, more than half (54%) of the respondents (n = 27) said they experienced an increasing trend in the occurrence of MTS in skipjack tuna, 29% said that it remained unchanged, and 14% reported a decreasing trend.

### 3.4. Contributing Factors and Consequences of Mushy Tuna Syndrome Incidence

**Figure 5:**
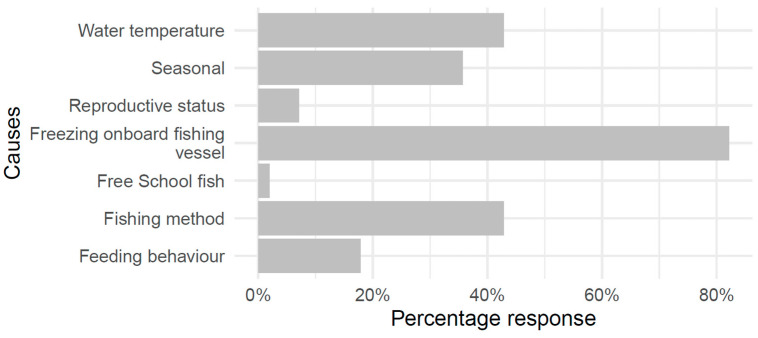
Industry perception of the potential causes of MTS (n = 28).

To understand industry perceptions about the likely causes of MTS, participants were asked to indicate which of seven potential causes they thought was the predominant cause for MTS (fishing method, onboard freezing, seasonal, free school fish, reproductive status, feeding behaviour, sea surface temperature, or other). Respondents (n = 28) noted more than one cause. On-board freezing in the fishing vessel was marked as a potential cause by 82% of the respondents, whereas sea surface temperature and fishing method were equal at 43%. A total of 39% of respondents indicated reproductive status as a potential cause, followed by seasonal factors (36%), feeding behaviour (29%), and free school catch (7%) (Figure 5). One of the participants stated that it was more common in fish weighing more than 3.4 kg: “Normally we find mushy in tuna weighing >3.4 kg”, and another said that “When the fishing is very good, the MTS is more common”.

Respondents who attributed seasonal factors to MTS incidence were asked to specify the months in which they observed the highest incidence of MTS. The respondent from Africa indicated the occurrence of MTS in the first half of the calendar year, whereas respondents from Asia and Oceania indicated the occurrence of MTS in the second half of the year. One of the respondents from Oceania commented that “MTS mostly occurs with school fish… …this can occur from October to Jan. The effects are further compounded when we get large sets (over 100 metric tonnes), and onboard freezing takes longer to complete”. A similar comment was also received from a respondent in South America, “Large fish and fish caught by free school [are] more likely to have MTS. Central Western Pacific seems to have a higher percentage MTS than Eastern Pacific”.

**Figure 6:**
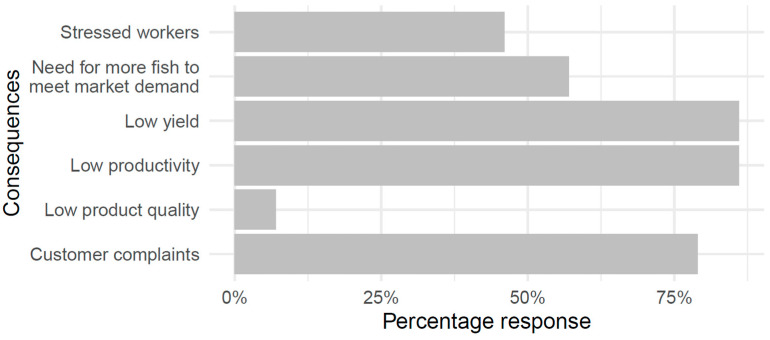
Consequence to business from the occurrence of MTS-affected fish (n = 28).

Participants were asked to indicate how their business was impacted by the occurrence of MTS-affected fish. Respondents reported that MTS causes low yield (86%), low productivity (86%), and more customer complaints (79%). Other causes indicated by the respondents included the need for more fish to meet market demand (57%), stressed workers (46%), and low product quality (7%) (Figure 6). Low product quality was also associated with customer complaints. One of the respondents noted that it was difficult to sell products with MTS, while another commented that buyers do not accept MTS-affected products.

Rejected batches were diverted for fishmeal production by 99% of participants who experienced MTS. One of the respondents mentioned that they “fine-tuned” their processing to accommodate MTS fish, and, depending on the texture of MTS loins, they changed the pack style or media from brine to an oil pack, or from steak to a chunk pack.

### 3.5. Nature of Skipjack Tuna Landings

Participants were asked to provide the percentage of the fishing methods that contributed to skipjack landing, and 89% (26/29) of participants who experienced MTS answered this question. Even though for the majority of the participants, fish caught in purse seine and brine-frozen on board contributed to their skipjack landings, for two of the participants, all of the skipjack they received were from vessels that used pole and line, and chilling on board before blast freezing at the factory—both of these participants noted MTS in their tuna. Participants were asked to indicate the total quantity of rejects caused by MTS and provide the quantity of rejects that came from fish caught using FADs (Fish Aggregating Devices). As only a few of the participants answered both questions, an analysis of rejects from FADs from the total rejects could not be calculated. However, it was noted that 38% (11/29) of those who experienced MTS had rejects from fish caught around FADs.

## 4. Discussion

Among all of the commercially important tuna species, skipjack accounts for more than half of the total volume of tuna landed globally, and are sold as canned tuna in nearly every region of the world [9,11], with 59% (n = 19) of the survey participants operating in Asia. Asia is the hub of the largest tuna processors, with Indonesia and the Philippines being among the top five tuna producers globally [5]. Nearly 30% (n = 9) of survey participants were from Oceania, a region where tuna is an important source of protein and export revenue, with tuna processing and fishing providing up to 25% of the GDP in several Pacific Island Countries and Territories [26] and the Western Central Pacific region contributing to nearly half of the world’s tuna fishery [9,27]. This study confirmed that MTS is predominantly reported in skipjack tuna. The results from this industry survey complement earlier reports of MTS incidence [20,21,22] and contribute to discussions regarding its implications for the tuna industry and its potential causes.

### 4.1. Extent of MTS in the Tuna Industry

Skipjack are epipelagic, inhabiting waters with temperatures from 14.7 °C to 30 °C, and have a cosmopolitan distribution in tropical and warm-temperature waters. They are widespread throughout the Indo-West Pacific but are absent from the eastern Mediterranean Sea and Black Sea [28]. The survey results revealed that MTS is prevalent across different geographical regions, indicating that it is not restricted to a particular area but distributed throughout the region where skipjack are found. Thus, it was not surprising to find MTS cases in the different geographic locations where skipjack is processed—confirming that MTS occurrence is global.

This study found that rejection rates and associated costs varied among participants, likely due to differences in the size of their operations and the volume of tuna they process. The cost of rejects reported ranged from less than 1000 USD to over 1 million USD. This study revealed that the median cost of rejection reported was more than 60,000 USD, and the average rejection rate was 1.8%. More than half of the respondents indicated an increasing trend in the occurrence of MTS, highlighting the need for further research into its causes. These findings highlight the significant financial impact of rejection rates on the industry. Participants in the Oceania region, which encompasses the Western Central Pacific Ocean (WCPO), reported particularly high costs related to MTS, as did Asia. It is worth noting that the WCPO accounts for more than half of the world’s tuna fishery, and skipjack tuna production in the area has increased from 1.0 million tonnes to over 1.8 million tonnes in the last two decades [6]. The revenue from tuna resources is vital as a source of income, employment, and food security for the region, and a food loss and waste scenario in the tuna value chain will have a substantial negative impact on the region.

### 4.2. Contributing Factors of Mushy Tuna Syndrome Incidence

Numerous extrinsic and intrinsic factors can influence the overall quality of fish, such as species characteristics, seasonal biological changes in the gonads and muscles, fishing grounds and fishing techniques, post-mortem factors, and processing conditions [29,30,31]. Based on the findings of the survey, there are several potential contributors to MTS (fishing method, onboard freezing, seasonal, free school fish, reproductive status, feeding behaviour, and sea surface temperature) that should be further examined.

Skipjack have been reported to have red muscle core temperatures as high as 10.7 °C above that of the surrounding water, which ranges from 14.7 °C to 30 °C [32]. Research into albacore tuna has shown that high body temperature when landed (26–30 °C) increases the initial rate of chemical reactions and enzymatic activity, contributing to decreased flesh quality before it is refrigerated [33]. Also, the more slowly the fish is frozen, the larger the ice crystals become, and large ice crystals damage the ultrastructure and concentrate the solutes in the meat, leading to alteration in biochemical reactions at the cellular level, providing greater potential for texture loss [34,35]. The muscle of the fish affected by MTS is mushier on the inner loin closest to the backbone and the stomach. This also highlights that delayed chilling during post-harvest handling could be another possible reason for further muscle degradation, as proteinases closest to the backbone are still active, even when the outer regions of the fish have started to cool down [36]. Temperature control during the various stages of thawing, handling, and thermal processing before precooking is therefore crucial in maintaining the texture quality of the canned tuna product [20].

Tunas are captured using various methods and types of gear, including pole-and-line bait boats, longline vessels, handlines, jig-boat trollers, and purse-seine vessels [14]. Fishing method and fishing set type (caught from free schools or using a Fish Aggregating device—FAD) affect fishing strategy, fishing vessel efficiency, onboard vessel handling, and time–temperature factors, which can all eventually impact fish quality, particularly the texture of the fish. It is important to note that onboard freezing in the fishing vessel was noted as a contributing factor of MTS by 82%, while 43% of the survey respondents indicated fishing method as a potential factor.

The personal observations and experiences of two of the authors (L.C.H, S.M.J.P) have indicated that when large volumes of fish are seine netted, by the time the last fish are loaded out of the nets into the chilling/freezing tanks, these fish are already dead from suffocation due to crowding in the nets; this acute ante mortem stress could result in the denaturation of proteins due to the rapid increase in muscle cellular lactic acid at high muscle temperatures. This denaturation of the proteins will decrease the water-binding capacity of the muscles, causing the meat to lose water. This aspect warrants further research, as does the impact of fishing method and fishing set type, mentioned above.

Noting that the report from the survey on the occurrence of MTS showed that MTS is not consistently seen throughout the year further confirms that there may be other factors that could trigger MTS prior to harvesting and freezing. Seasonal trends in the occurrence of MTS raise the question of the effect of environmental and biological conditions on the texture of the muscle post-harvest and post-cooking. For most participants, the incidence of MTS was not the same in all years. Kaneko and Nakamura [22] noted the coincidence of MTS with the El Nino event, when there is a rise in surface water temperature in the Central and Eastern Pacific Ocean, indicating the role of temperature and changing oceanic conditions in MTS.

### 4.3. Consequence of Mushy Tuna Syndrome Incidence to Business

For the canning industry, which is the main pathway to consumers for most of the world’s tuna catch [37], texture is one of the most important quality factors, and is driven by consumer expectations of a high-quality product [38]. Achieving a higher yield—meaning more canned product per tonne of processed tuna—is a top priority, while also ensuring good quality canned meat. The survey results show that lower yield and lower productivity were followed by customer complaints as the main consequences of MTS, which affects the textural quality of tuna meat. Processed meat that is no longer suitable for processing is downgraded to fishmeal production and more fish need to be caught and processed to meet market demands. A total of 46% of the respondents noted stressed workers as one of the consequences, due to the drive to meet customer expectations of high-quality tuna product. Overall, it is evident that MTS is of consequence to businesses that process skipjack tuna.

## 5. Conclusions

We have made the first attempt to quantify the prevalence and severity of Mushy Tuna Syndrome globally. We found both a high prevalence (97% of the respondents noting that they have had MTS in their skipjack tuna) and business costs ranging from less than 1000 USD to over 1 million USD around the world due to rejections and downgrading, lower yield, low productivity, and customer complaints. The higher levels of rejection could affect sustainability, as the industry needs more of the catch to process fish for the same contracted volumes of the final product.

The environmental conditions, such as sea surface temperature, reproductive stage, the behaviour of fish while feeding, and the potential impact of the harvesting techniques and subsequent freezing methods, which could lead to stressful muscular activity following capture and eventually affects the pH of the fish, are all potential extrinsic and intrinsic factors that could cause MTS. Further research is needed to understand the biochemical basis of MTS and understand the integration of disparate areas of study to forecast—based on the environmental and fish body conditions, harvest, and handling methods—the occurrence of MTS, particularly under climate change conditions. An increased understanding of the contribution of these factors will be vital to the development of forecast tools that will benefit the fishers and processors by allowing them to adjust harvest strategies, fishing practices, and processing practices, through the identification of conditions that are likely to be associated with MTS. A cost-effective, rapid, non-invasive technique to identify fish that have a high probability of displaying MTS would also be useful, as it would enable processors to divert such fish to an appropriate product line. This would reduce fish waste and increase economic efficiency and fishery sustainability.

With only seven years to go in ‘the Ocean Decade’ for achieving the Sustainable Development Goals set by the United Nations, time is running out to achieve the outlined 50% reduction in fish loss and waste. Investment is needed to help the industry manage the problem and to reduce food loss. One clear priority would be to identify the proximate biological, physical, and bio-chemical factors associated with Mushy Tuna Syndrome so that capture and processing strategies can be altered to increase economic efficiency and reduce food loss and waste.

## Figures and Tables

**Table 1:** Survey participant’s characteristics (n = 32).

Characteristic		(%)
*Region*		
Asia		59%
Oceania		28%
South America		9%
Africa		3%
*Nature of business*		
Canned tuna production		67%
Frozen precooked tuna loins		81%
Whole round frozen		56%
*Production capacity*		
>100 MT tuna/day, >2300 workers		59%
<100 MT tuna/day, >500 workers		31%
*Species processed*		
Skipjack		97%
Yellowfin		97%
Bigeye		66%
Albacore		41%
Bonito		13%
Tonggol		6%

## Data Availability

The data that support the findings of this study are not publicly available due to privacy or ethical restrictions.
